# Preparative Separation and Purification of Trichothecene Mycotoxins from the Marine Fungus *Fusarium* sp. LS68 by High-Speed Countercurrent Chromatography in Stepwise Elution Mode

**DOI:** 10.3390/md16020073

**Published:** 2018-02-24

**Authors:** Yong Liu, Xuezhen Zhou, C. Benjamin Naman, Yanbin Lu, Lijian Ding, Shan He

**Affiliations:** 1 Li Dak Sum Yip Yio Chin Kenneth Li Marine Biopharmaceutical Research Center, Ningbo University, Ningbo 315211, China; 18892618896@163.com (Y.L.); zhouxuezhen@nbu.edu.cn (X.Z.); bnaman@gmail.com (C.B.N.); 2 Key Laboratory of Marine Biotechnology of Zhejiang Province, Ningbo University, Ningbo 315211, Zhejiang, China; 3 Center for Marine Biotechnology and Biomedicine, Scripps Institution of Oceanography and Skaggs School of Pharmacy and Pharmaceutical Sciences, University of California, San Diego, La Jolla, CA 92093, USA; 4 Institute of Seafood, Zhejiang Gongshang University, Hangzhou 310012, China; luyanbin@zjgsu.edu.cn

**Keywords:** trichothecene mycotoxins, roridin, verrucarin, high-speed countercurrent chromatography, preparative separation, stepwise elution

## Abstract

The contamination of foods and animal feeds with trichothecene mycotoxins is a growing concern for human and animal health. As such, large quantities of pure trichothecene mycotoxins are necessary for food safety monitoring and toxicological research. A new and effective method for the purification of trichothecene mycotoxins from a marine fungus, *Fusarium* sp. LS68, is described herein. Preparative high-speed countercurrent chromatography (HSCCC) was utilized for the scalable isolation and purification of four trichothecene mycotoxins for the first time in stepwise elution mode, with a biphasic solvent system composed of hexanes–EtOAc–CH_3_OH–H_2_O (6:4:5:5, *v*/*v*/*v*/*v*) and (8.5:1.5:5:5,*v*/*v*/*v*/*v*). This preparative HSCCC separation was performed on 200 mg of crude sample to yield four trichothecene mycotoxins, roridin E (**1**), roridin E acetate (**2**), verrucarin L acetate (**3**), and verrucarin J (**4**) in a single run, with each of >98% purity. These compounds were identified by MS, ^1^H NMR, ^13^C NMR, and polarimetry. The results demonstrate an efficient HSCCC method for the separation of trichothecene mycotoxins, which can be utilized to produce pure commercial and research standards.

## 1. Introduction

According to the Food and Agriculture Organization of the United Nations, about 25% of the food crops in the world are contaminated with mycotoxins, and these have adverse effects on humans, animals, and crops that result in serious illnesses and economic losses [1]. Among the major mycotoxins produced by *Fusarium* species, trichothecenes are pathogenic to important agricultural crops and foods [2], and these lead to serious economic losses by reducing yields and overall quality of North American agricultural products [3,4]. For example, *Fusarium* head blight disease, caused by trichothecene mycotoxins, has been shown to have contaminated cereals and grains [5,6].

Trichothecenes are a group of tetracyclic sesquiterpene mycotoxins that are produced by various fungi from the order *Hypocreales*, including those of the genera *Fusarium*, *Myrothecium*, *Verticimonosporium*, *Stachybotrys*, *Trichoderma*, *Trichothecium*, *Cephalosporium*, and *Cylindrocarpon* [7,8,9,10,11]. These molecules are potent phytotoxins, and act as the virulence factors of pathogenic fungi on sensitive infected host plants, particularly wheat and barley [12,13,14]. The unfortunate contamination of botanical dietary supplements with mycotoxins has also been reported recently, representing a significant risk to public health [15]. One obstacle to the further investigation of these important topics has been the scarce quantity of pure compounds from the trichothecene class. Therefore, the preparative separation of high purity trichothecene mycotoxins is of great interest. At present, trichothecene mycotoxins are isolated from fungal extracts by conventional methods, such as macroporous resin separation followed by silica gel column chromatography. These methods can be considered costly, laborious, and time-consuming, and furthermore result in substantial sample loss due to irreversible adsorption to the silica [16,17]. It is therefore important to develop rapid and efficient methods for the separation and purification of trichothecene mycotoxins.

**Figure 1:**
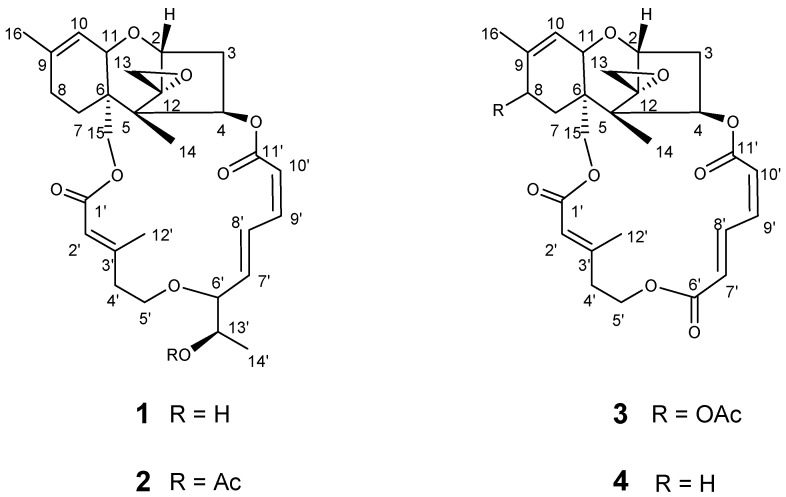
Structures of roridin E (**1**), roridin E acetate (**2**), verrucarin L acetate (**3**), verrucarin J (**4**).

High-speed countercurrent chromatography (HSCCC) is a technique that uses liquid-liquid partition chromatography to improve recovery rates and efficiency, and which eliminates irreversible adsorption by solid support media [17,18,19]. HSCCC has been widely used for the preparative separation and purification of natural products, and this is a scalable technology [20,21]. Disclosed herein is the first preparative isolation and purification of trichothecene mycotoxins by HSCCC in stepwise elution mode, allowing for the purification of several compounds in one run. The trichothecene mycotoxins roridin E (**1**), roridin E acetate (**2**), verrucarin L acetate (**3**), and verrucarin J (**4**) were thus isolated from an epiphytic fungus, *Fusarium* sp. LS68, which was derived from the sponge *Haliclona* sp. The structures of **1**–**4** are presented in Figure 1.

## 2. Results and Discussion

### 2.1. HPLC Analysis of the Crude Extract

**Figure 2:**
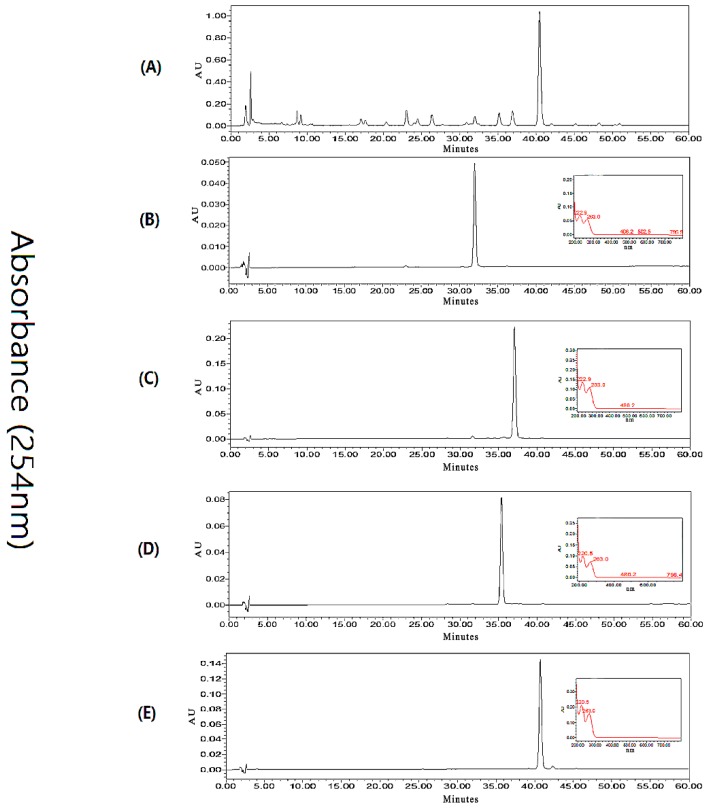
Representative analytical HPLC chromatograms. (**A**) Crude extract of *Fusarium* sp; (**B**) refined peak 1 from HSCCC, corresponding to compound **1**; (**C**) refined peak 2 from HSCCC, corresponding to compound **3**; (**D**) refined peak 3 from HSCCC, corresponding to compound **2**; (**E**) refined peak 4 from HSCCC, corresponding to compound **4**. For B–D, UV profiles of the major peak are inset at 254 nm.

An HPLC method was developed to ensure the baseline separation of the target compounds and impurities. Different solvent systems, flow rates, detection wavelengths, and column temperatures were screened. The components of interest, **1**–**4**, were satisfactorily separated by a CH_3_CN/H_2_O gradient (flow rate 0.8 mL/min, 25–75% CH_3_CN from 0–60 min) when the column temperature was set at 25 °C. The HPLC chromatogram of the crude extract is shown in Figure 2, Panel A. The same method was used to analyze HSCCC method development samples, and later HSCCC eluents (Figure 2, Panels B–E), for fraction pooling.

### 2.2. Optimization of Suitable HSCCC Solvent System

In order to efficiently separate and purify target compounds, the selection of an optimal biphasic solvent system is critical for preparative HSCCC. According to the “golden rules of HSCCC”, satisfactory partition coefficient *K*_D_ values should meet some basic requirements: (1) the target compounds should each be in the range of 0.5 ≤ *K*_D_ ≤ 2.0; (2) each set of two target compounds should have the separation factor *α* > 1.5, where *α* is the ratio of the two *K*_D_ values (*α* = *K*_D_^1^/*K*_D_^2^, for *K*_D_^1^ > *K*_D_^2^); and (3) higher retention of the stationary phase will provide better peak resolution [22]. Thus, preliminary studies were carried out to examine the *K*_D_ values of the trichothecene mycotoxins **1**–**4** in different hexanes–EtOAc–CH_3_OH–H_2_O solvent systems of different volume ratios by liquid-liquid partitioning and HPLC analysis. The *K*_D_ values for **1**–**4** in the various solvent systems tested are shown in Table 1.

No single suitable biphasic solvent system was found that had *K*_D_ values between 0.5 and 2 for each of compounds **1**–**4** at the same time. To overcome this challenge, a stepwise HSCCC elution mode was chosen in order to separate compounds with significantly different *K*_D_ values in a single run. This technique had been successfully applied previously by some research groups [23,24].

The use of the “HeMWat”, or hexanes–EtOAc–CH_3_OH–H_2_O, solvent system at volumetric ratios of 1:1:1:1 and 5.5:4.5:5:5 both afforded larger *K*_D_ values unsuitable for the separation of the trichothecene mycotoxins investigated. However, the biphasic solvent system of hexanes–EtOAc–CH_3_OH–H_2_O at a volumetric ratio of 6.5:3.5:5:5 yielded acceptable *K*_D_ values of compounds **1**–**3** between 0.5 and 2, although compounds **2** and **3** had an unsuitable separation factor *α* < 1.5. However, the biphasic hexanes–EtOAc–CH_3_OH–H_2_O solvent system at a volume ratio of 6:4:5:5 led to the observation of the *K*_D_ values between 0.5 and 2 for compounds **1**, **2**, and **3**, and each pair had a separation factor *α* > 1.5. Nevertheless, this solvent system offered an unsuitable *K*_D_ value (2.58) for compound **4**. To overcome this problem, the hexanes–EtOAc–CH_3_OH–H_2_O 8.5:1.5:5:5 solvent system was selected for the second part of the HSCCC run, since it produced a suitable *K*_D_ value of 1.12 for compound **4**, and it would only be used in stepwise mode after the elution of **1**–**3**.

### 2.3. Stepwise HSCCC Separation

**Figure 3:**
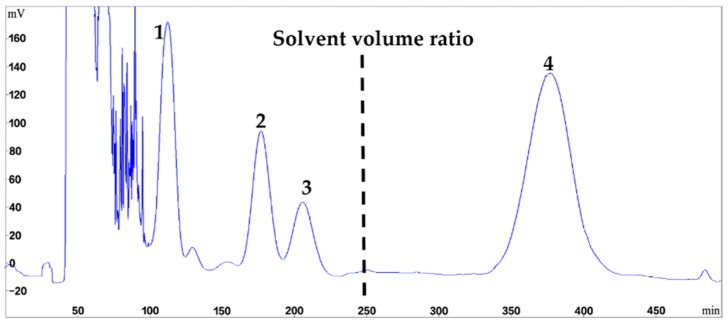
HSCCC chromatogram of the crude extract from *Fusarium* sp. LS68 using stepwise elution with solvent systems A and B. The dotted line represents the time at which the solvent system was switched from A to B. Solvent system A: hexanes–EtOAc–CH_3_OH–H_2_O (6:4:5:5, *v*/*v*/*v*/*v*), solvent system B: hexanes–EtOAc–CH_3_OH–H_2_O (8.5:1.5:5:5, *v*/*v*/*v*/*v*); stationary phase: upper phase of solvent system A; mobile phase: lower aqueous phase of solvent system A and B; column capacity, 350 mL; rotation speed, 800 rpm; column temperature, 25 °C; flow rate, 2.0 mL/min; detection, 254 nm; sample injected, 200 mg in 6 mL biphasic solution; retention of the stationary phase, 63%; peak identification: roridin E (**1**), roridin E acetate (**2**), verrucarin L acetate (**3**), verrucarin J (**4**).

The optimized stepwise elution method was applied for the direct preparative HSCCC separation of 200 mg of crude *Fusarium* extract in a single run. As shown in Figure 3, the separation initiated with the solvent system of hexanes–EtOAc–CH_3_OH–H_2_O 6:4:5:5, and the upper phase was used as the stationary phase while the lower phase was used as the mobile phase in the head-to-tail elution mode. Retention of the stationary phase was 63%, and after peaks 1–3 eluted at 102, 180, and 205 min, respectively, the mobile phase was switched to the lower phase of the solvent system (8.5:1.5:5:5) at 250 min. Peak 4 was well resolved, and eluted at 350 min. Timewise fractions were collected from the HSCCC separation and pooled after evaluation by HPLC, to yield compound **1** (11.3 mg), compound **2** (7.7 mg), compound **3** (4.7 mg), and compound **4** (21.3 mg). Each compound isolated had a purity of >98% based on UV peak integrations (Figure 2, Panels B–E). The chromatogram of HSCCC separation is shown in Figure 3.

### 2.4. Structural Identification

The structural identification of **1**–**4** was conducted by HRESI-MS, ^1^H and ^13^C NMR analyses, as well as specific rotation data, and these were compared with literature values. Accordingly, the molecules isolated were identified as roridin E (**1**) [25], roridin E acetate (**2**) [26], verrucarin L acetate (**3**) [27], and verrucarin J (**4**) [27] (see Supplementary Materials).

## 3. Materials and Methods

### 3.1. Reagents and Materials

All solvents used for HSCCC were of analytical grade (Huadong Chemicals, Hangzhou, China). Reverse osmosis Milli-Q water (18 M) (Millipore, Bedford, MA, USA) was used for all solutions and dilutions. Methanol used for HPLC analyses was of chromatographic grade and purchased from Anpel Laboratory Technologies (Shanghai, China). The CDCl_3_ used for NMR analyses was purchased from Tenglong Weibo Technology (Qingdao, China).

### 3.2. Fungal Material

The marine fungi *Fusarium* sp. LS68, was isolated and cultured from a *Halicloma* sp. sponge collected from Linshui, Hainan Province, China. It was identified as *Fusarium* sp. according to the morphological and molecular (ITS rDNA sequence) analyses (GenBank accession number EU860057, 99% similarity). A voucher specimen was deposited at the School of Marine Sciences, Ningbo University (Ningbo, China).

### 3.3. Apparatus

HSCCC was carried out using a model TBE-300C high-speed countercurrent chromatograph (Tauto Biotech Co. Ltd., Shanghai, China), containing a self-balancing three-coil centrifuge rotor equipped with three preparative multilayer coils and a total capacity of 320 mL. The internal diameter of PTFE (Polytetrafluoroethylene) tubing was 1.9 mm. The apparatus maximum rotational speed is 1000 rpm and has a 20 mL manual sample loop. The revolution radius was 5 cm and the β value of the multilayer coil varied from 0.5 at the internal terminal to 0.8 at the external terminal. An integrated TBP 5002 (Tauto Biotech Co. Ltd.) was used to pump the two-phase HSCCC solvent system, and the UV absorbance of the effluent was measured at 254 nm by a UV 1001 detector (Shanghai Sanotac Scientific Instruments Co. Ltd., Shanghai, China). A DC-0506 constant temperature regulator (Tauto Biotech Co. Ltd.) was used to control the temperature during HSCCC. An N2000 data analysis system (Institute of Automation Engineering, Zhejiang University, Hangzhou, China) was employed for HSCCC data collection and analysis. The analytical HPLC equipment was an Alliance 2695 equipped with a model 2998 diode array detector and Empower System (Waters Co., Milford, MA, USA). NMR experiments were carried out using a DirectDrive2 600 MHz NMR spectrometer (Agilent, Santa Clara, CA, USA) at 25 °C and Auto Triple Resonance (Agilent, Santa Clara, CA, USA). HRESIMS data were measured using a Waters Q-TOF Premier LC/MS spectrometer (Waters Co., Milford, MA, USA). Column chromatography was conducted with silica gel (200–300 mesh, Qingdao Marine Chemical Inc. Qingdao, China). The UV spectra were recorded on an NADE Evolution 201 spectrophotometer (Waters Co., Milford, CT, USA). Optical rotations were measured on an Autopol VI (Rudolph Research Analytical, Hackettstown, NJ, USA).

### 3.4. Culturing and Extraction

The fungus *Fusarium* sp. LS68 was cultivated in a seawater-based potato dextrose broth (PDB) medium and incubated on a rotary shaker at 150 rpm and 25 °C for 14 days. The fermentation broth (30 L) was extracted with EtOAc (3 × 30 L). The organic solvent partitions were combined and evaporated under reduced pressure at 40 °C to yield a crude extract (30 g). This sample was stored in a refrigerator at 4 °C until the subsequent HSCCC separation.

### 3.5. Determination of Partition Coefficients (K_D_)

In order to determine the appropriate solvent systems for optimal partition coefficients (*K*_D_), various solvent combinations of hexanes–EtOAc–CH_3_OH–H_2_O were attempted. A 2-mg sample of crude extract was added to a 10 mL test tube along with 8 mL of an experimental biphasic solvent system. The test tube was then capped and shaken vigorously for 3 min, to allow the distribution of the extract between the two phases. After reaching equilibration, equal aliquots of each phase (10 μL) were analyzed by HPLC-DAD to determine the partition coefficient (*K*_D_) of each target compound. The *K*_D_ values were defined as the integrated peak area of each target compound in the upper phase divided by that in the lower phase, as observed at 254 nm.

### 3.6. HSCCC Separation

The biphasic solvent systems of hexanes–EtOAc–CH_3_OH–H_2_O (6:4:5:5 and 8.5:1.5:5:5) were selected for the HSCCC separation. A 200 mg sample of crude extract was dissolved in 6 mL of each phase of a solvent mixture consisting of equal volumes of both upper and lower phases for preparative HSCCC separation. In the single run, the upper (stationary) phase was pumped into and filled the coil column of TBE-300C (320 mL, Tauto Biotech Co. Ltd., Shanghai, China) by a constant flow at 10.0 mL/min. The column was then rotated at a speed of 800 rpm, and the lower (mobile) phase was pumped into the column in the “head to tail” elution mode at 2.0 mL/min. When the mobile phase began to elute at the tail outlet, the hydrodynamic equilibrium state of the two phases was established. Subsequently, the sample solution (6 mL) containing 200 mg of crude extract was injected through the sample port. After 250 min, the pump was stopped and then the lower phase of hexanes–EtOAc–CH_3_OH–H_2_O (8.5:1.5:5:5) was pumped into the column as the new mobile phase at 2.0 mL/min. The separation temperature was controlled at 25 °C. The effluent was continuously monitored with a UV detector (Shanghai Sanotac Scientific Instruments Co. Ltd., Shanghai, China) at 254 nm. After the separation procedure, the solvents in the column were completely eluted and collected. Fractions were collected near 110 min, 180 min, 210 min, and 380 min, respectively, and analyzed by HPLC to allow for fraction pooling. 

### 3.7. HPLC Analysis and Identification of the Peaks

Each fraction generated by preparative HSCCC was evaluated by HPLC, using a 150 mm × 4.6 mm i.d., 5 μm, YMC-Pack ODS-A column (Waters Co., Milford, CT, USA) that was maintained at 25 °C. The samples were separated with a CH_3_CN/H_2_O gradient (flow rate 0.8 mL/min, 25–75% CH_3_CN from 0–60 min). The effluent was continuously monitored by a UV detector at 220 nm and 254 nm. Fractions that showed only one peak in the chromatogram were respectively pooled together to yield compounds **1** (11.3 mg, the *t*_R_(HPLC) 32 min, *t*_R_(HSCCC) 102–120 min), **2** (7.7 mg, *t*_R_(HPLC) 37 min, *t*_R_(HSCCC) 180–195 min), **3** (4.7 mg, *t*_R_(HPLC) 35 min, *t*_R_(HSCCC) 205–220 min), and **4** (21.3 mg, *t*_R_(HPLC) 40 min, *t*_R_(HSCCC) 350–400 min). Compounds **1**–**4** were identified as roridin E (**1**) [25], roridin E acetate (**2**) [26], verrucarin L acetate (**3**) [27], and verrucarin J (**4**) [27] by comparison of their UV, ^1^H and ^13^C NMR, and specific rotation data with literature values. The purity levels of these samples were determined by peak area percentage after the automated integration of each chromatogram.

## 4. Conclusions

Trichothecenes are worldwide plant toxins that cause enormous yield losses in major economic crops. Some researchers have highlighted the need to decontaminate mycotoxins in foods and feeds [28]. However, investigations of trichothecene phytotoxicity are limited due to the difficulty and expense of obtaining trichothecene mycotoxins in high purity and sufficient quantity. In this study, the first efficient preparative separation of four trichothecene mycotoxins from the marine fungi *Fusarium* sp. LS68 by a stepwise HSCCC elution method was developed. The purities of the trichothecene mycotoxins isolated, **1**–**4**, were >98%. Therefore, the stepwise HSCCC method is an efficient technique for the isolation of high purity trichothecene mycotoxins. This method is industrially scalable with the size of the HSCCC instrumentation, and may provide a means to obtain pure trichothecene mycotoxins in the quantities needed for broader biological examinations and the generation of analytical standards for the agricultural food, supplement, and feedstock industries.

## Figures and Tables

**Table 1:** Partition coefficients (*K*_D_) of compounds **1**–**4** in several hexanes–EtOAc–CH_3_OH–H_2_O solvent systems tested for high-speed countercurrent chromatography (HSCCC) separation.

Solvent System (*v*/*v*) Hexanes–EtOAc–CH_3_OH–H_2_O	*K*_D_			
	Compound 1	Compound 2	Compound 3	Compound 4
1:1:1:1	2.08	2.61	2.33	10.58
5.5:4.5:5:5	0.93	2.25	2.14	4.73
6:4:5:5	0.61	1.83	1.26	3.32
6.5:3.5:5:5	0.37	1.43	1.12	2.58
7:3:5:5	0.45	1.25	0.80	3.21
7.5:2.5:5:5	0.36	1.10	0.68	2.73
8:2:5:5	0.19	0.58	0.54	1.87
8.5:1.5:5:5	0.11	0.37	0.32	1.12
9:1:5:5	0.10	0.30	0.25	0.83

## Supplementary Materials

The following are available online at http://www.mdpi.com/1660-3397/16/2/73/s1, Figure S1a, ^1^H NMR spectrum of compound **1** (CDCl_3_, 600 MHz); Figure S1b, ^13^C NMR spectrum of compound **1** (CDCl_3_, 150 MHz); Figure S1c, HRESIMS spectrum of compound **1**; Figure S1d, UV spectrum of compound **1**; Figure S2a, ^1^H NMR spectrum of compound **2** (CDCl_3_, 600 MHz); Figure S2b, ^13^C NMR spectrum of compound **2** (CDCl_3_, 150 MHz); Figure S2c, HRESIMS spectrum of compound **2**; Figure S2d, UV spectrum of compound **2**; Figure S3a, ^1^H NMR spectrum of compound **3** (CDCl_3_, 600 MHz); Figure S3b, ^13^CNMR spectrum of compound **3** (CDCl_3_, 150 MHz); Figure S3c, HRESIMS spectrum of compound **3**; Figure S3d, UV spectrum of compound **3**; Figure S4a, ^1^H NMR spectrum of compound **4** (CDCl_3_, 600 MHz); Figure S4b, ^13^CNMR spectrum of compound **4** (CDCl_3_, 150 MHz); Figure S4c, HRESIMS spectrum of compound **4**; Figure S4d, UV spectrum of compound **4**; Table S1, NMR data of compound **1** in CDCl_3_; Table S2, NMR data of compound **2** in CDCl_3_; Table S3, NMR data of compound **3** in CDCl_3_; Table S4, NMR data of compound **4** in CDCl_3_; Table S5, Specific rotation values of compounds **1**–**4**; Table S6, UV values of compounds **1**–**4**.
