# Supplementary material for: Preparative Separation and Purification of Trichothecene Mycotoxins from the Marine Fungus Fusarium sp. LS68 by High-Speed Countercurrent Chromatography in Stepwise Elution Mode
Source: Mar Drugs. 2018 Feb 24;16(2):73. doi: 10.3390/md16020073 (PMC5852501; doi:10.3390/md16020073)
Supplement: Supplementary file 1 [file marinedrugs-16-00073-s001.pdf]

Supplementary Materials

Preparative Separation and Purification of Trichothecene Mycotoxins from the Marine Fungus *Fusarium* sp. LS68 by High-Speed Counter-current Chromatography in Stepwise Elution Mode

Yong Liu [1], Xuezhen Zhou [1], C. Benjamin Naman [1,2,3], Yanbin Lu [4], Lijian Ding [1,2,*] and Shan He [1,2,*]

[1] Li Dak Sum Yip Yio Chin Kenneth Li Marine Biopharmaceutical Research Center, Ningbo University, Ningbo 315211, China; E-Mails: 18892618896@163.com (Y.L.); zhouxuezhen@nbu.edu.cn (X.Z); dinglijian@nbu.edu.cn (L.D.).

[2] Key Laboratory of Marine Biotechnology of Zhejiang Province, Ningbo University, Ningbo, Zhejiang 315211, China;

[3] Center for Marine Biotechnology and Biomedicine, Scripps Institution of Oceanography and Skaggs School of Pharmacy and Pharmaceutical Sciences, University of California, San Diego, La Jolla, CA 92093, USA

[4] Institute of Seafood, Zhejiang Gongshang University, Hangzhou 310012, China

* Correspondence: heshan@nbu.edu.cn (S.H.), dinglijian@nbu.edu.cn (L.D.); Tel.: +86-574-8760-0458 (S.H.).

Table of Contents **page**

Figure S1a. ^{1}H NMR spectrum of compound **1** ($CDCl_3$, 600 MHz) 3

Figure S1b. ^{13}C NMR spectrum of compound **1** ($CDCl_3$, 150 MHz) 3

Figure S1c. HRESIMS spectrum of compound **1** 4

Figure S1d. UV spectrum of compound **1** 4

Figure S2a. ^{1}H NMR spectrum of compound **2** ($CDCl_3$, 600 MHz) 5

Figure S2b. ^{13}C NMR spectrum of compound **2** ($CDCl_3$, 150 MHz) 5

Figure S2c. HRESIMS spectrum of compound **2** 6

Figure S2d. UV spectrum of compound **2** 6

Figure S3a. ^{1}H NMR spectrum of compound **3** ($CDCl_3$, 600 MHz) 7

Figure S3b.^{13}CNMR spectrum of compound **3** ($CDCl_3$, 150 MHz) 7

Figure S3c. HRESIMS spectrum of compound **3** 8

Figure S3d. UV spectrum of compound **3** 8

Figure S4a. ^{1}H NMR spectrum of compound **4** ($CDCl_3$, 600 MHz) 9

Figure S4b.^{13}CNMR spectrum of compound **4** ($CDCl_3$, 150 MHz) 9

Figure S4c. HRESIMS spectrum of compound **4** 10

Figure S4d. UV spectrum of compound **4** 10

Table S1. NMR data of compound **1** in $CDCl_3$ 11

Table S2. NMR data of compound **2** in $CDCl_3$ 12

Table S3. NMR data of compound **3** in $CDCl_3$ 13

Table S4. NMR data of compound **4** in $CDCl_3$ 14

Table S5. Specific rotation values of compounds **1–4** 15

Table S6. UV values of compounds **1–4**. 15

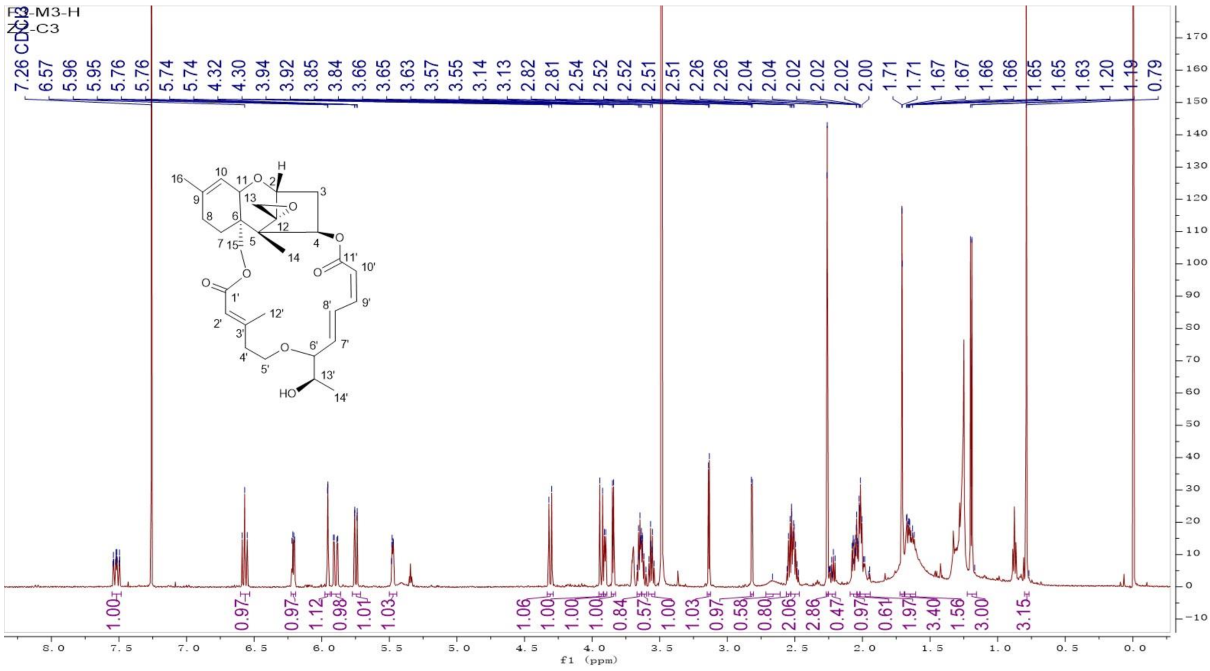

Figure S1a. ^{1}H NMR spectrum of compound **1** ($CDCl_3$, 600 MHz)

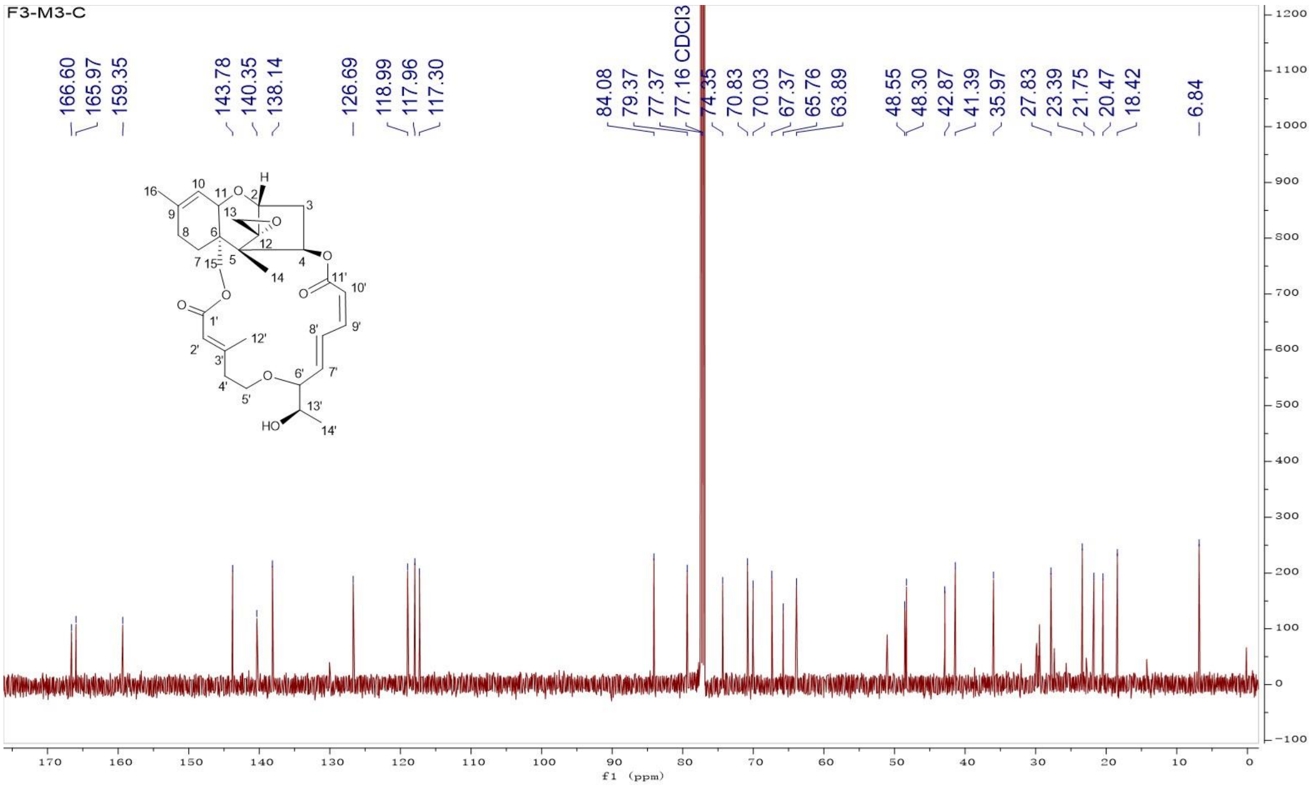

Figure S1b. ^{13}C NMR spectrum of compound **1** ($CDCl_3$, 150 MHz)

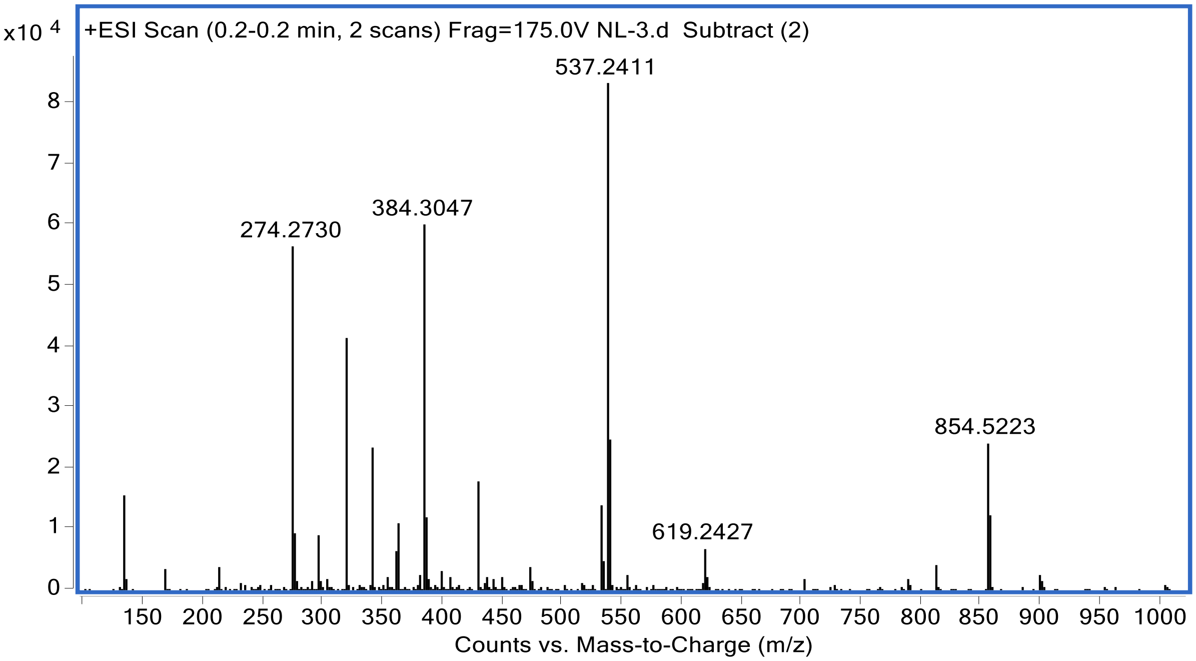

Figure S1c. HRESIMS spectrum of compound **1**

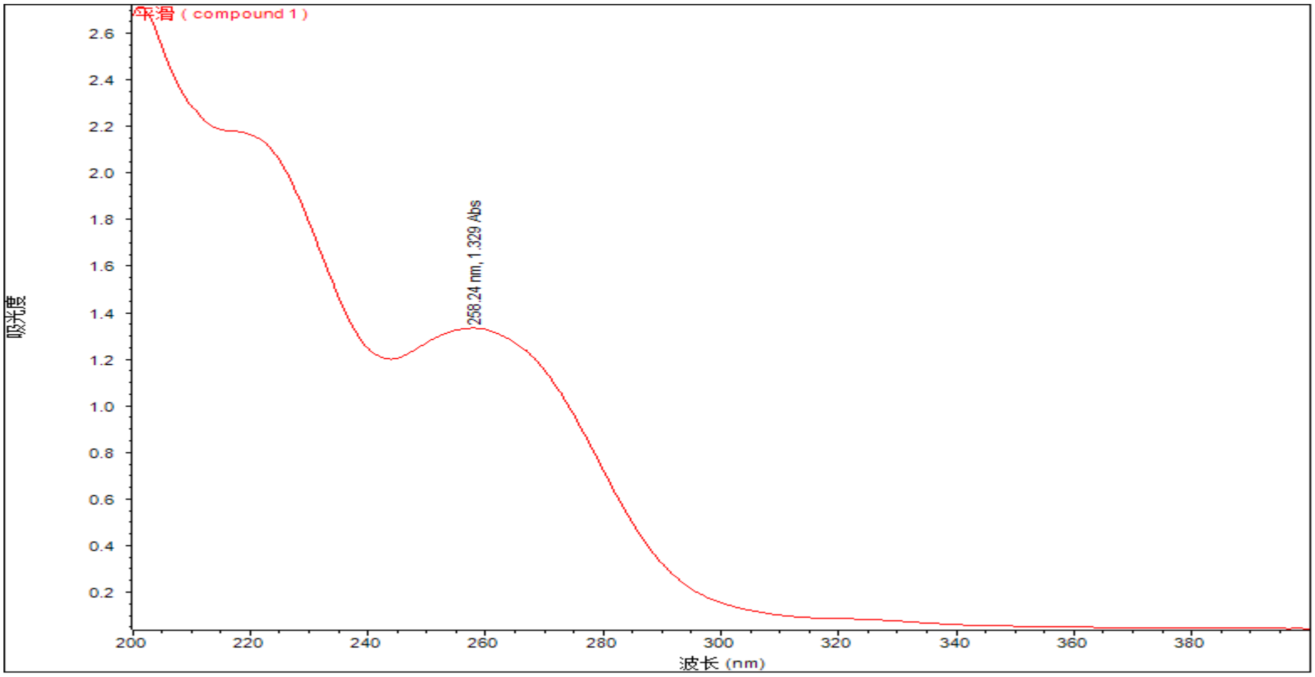

Figure S1d. UV spectrum of compound **1**

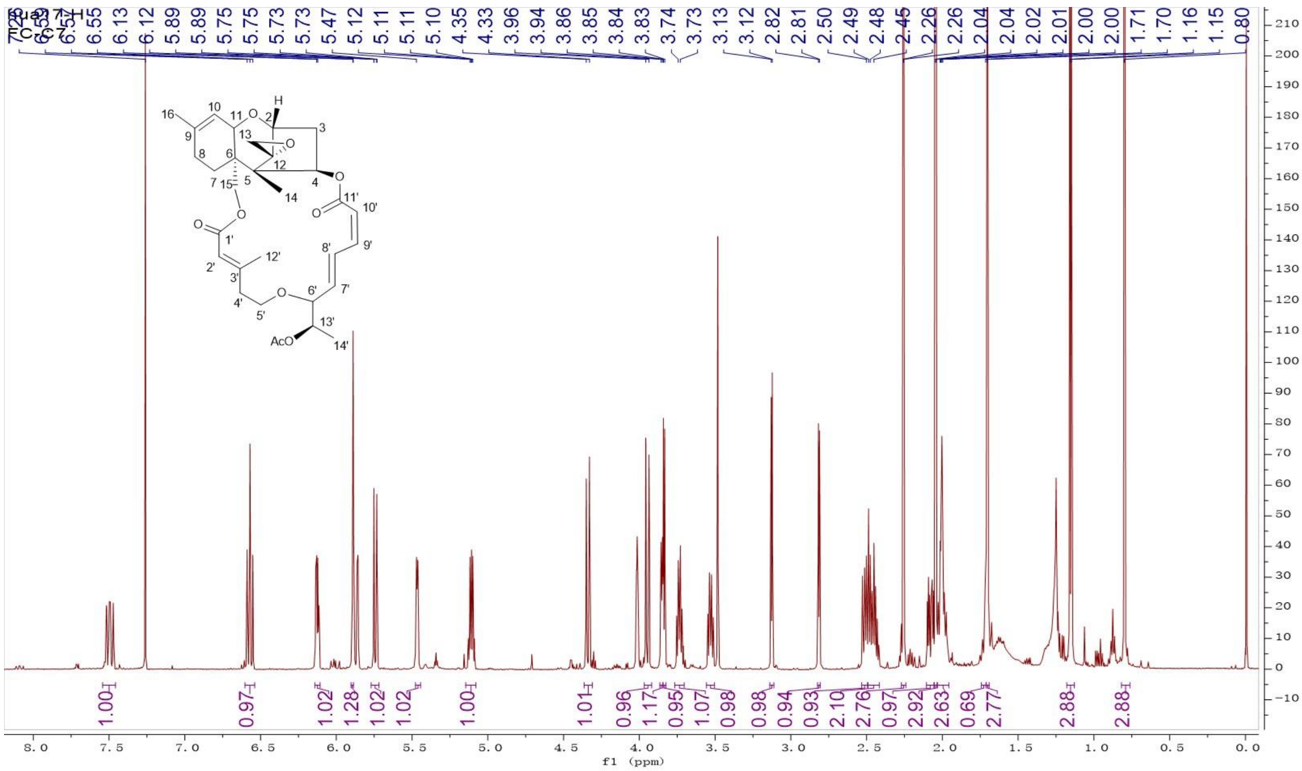

Figure S2a. ^{1}H NMR spectrum of compound **2** ($CDCl_3$, 600 MHz)

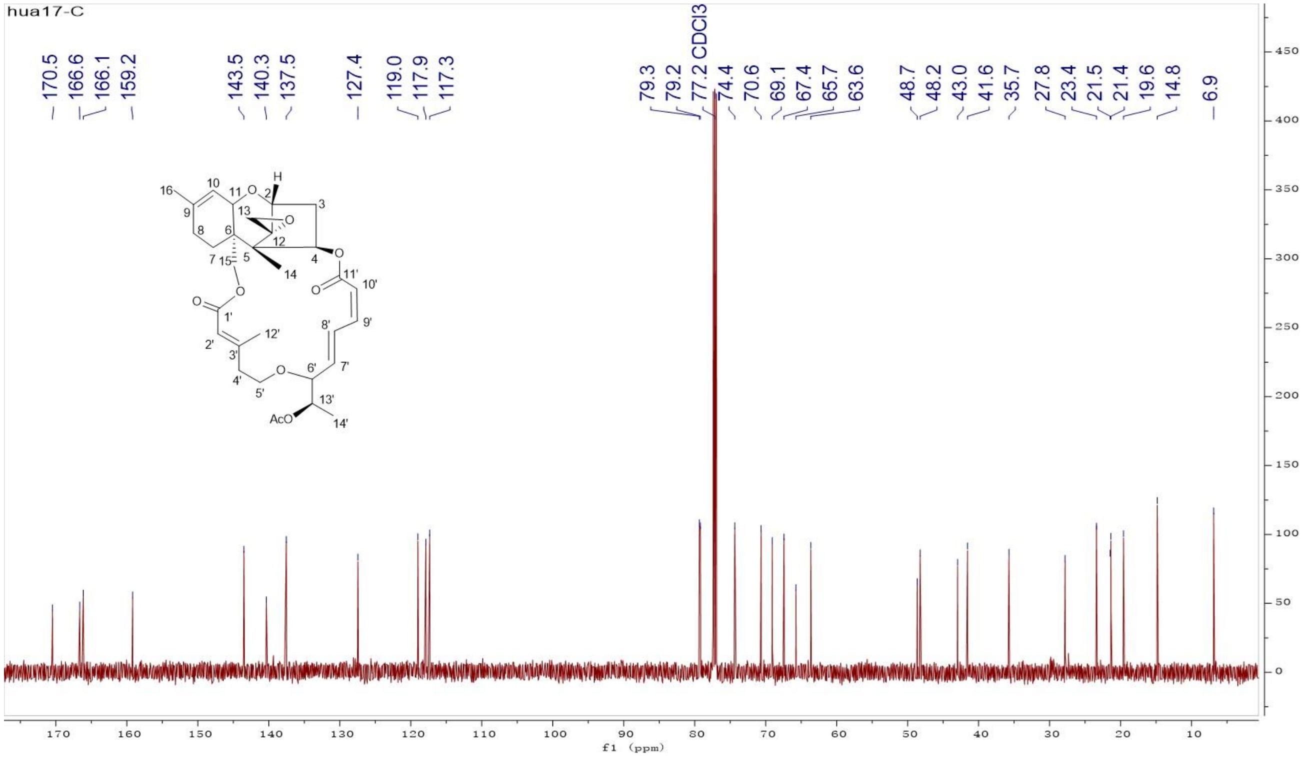

Figure S2b. ^{13}C NMR spectrum of compound **2** ($CDCl_3$, 150 MHz)

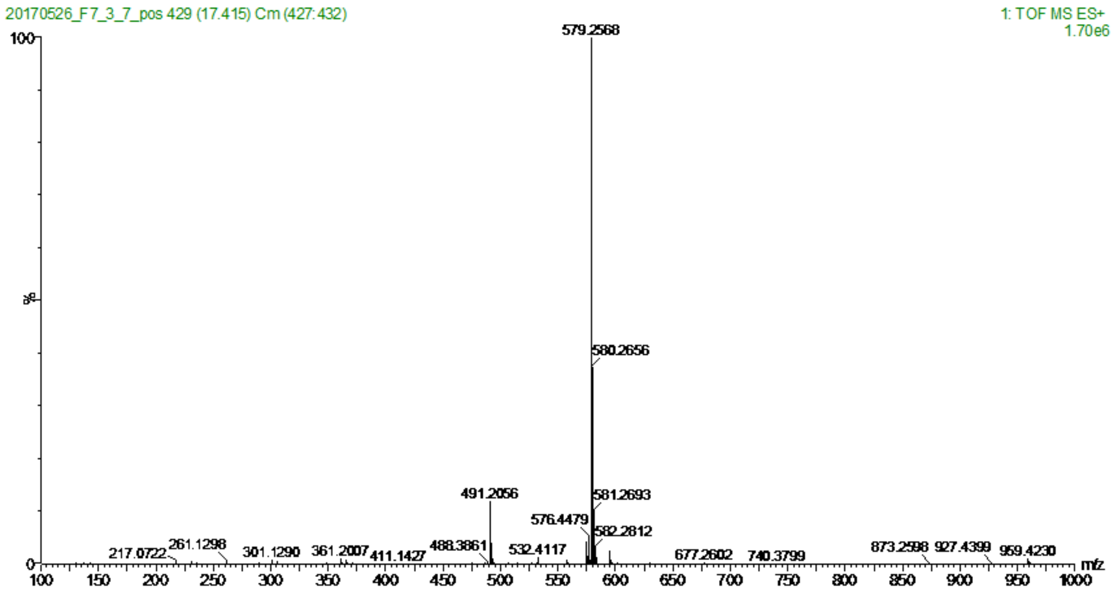

Figure S2c. HRESIMS spectrum of compound **2**

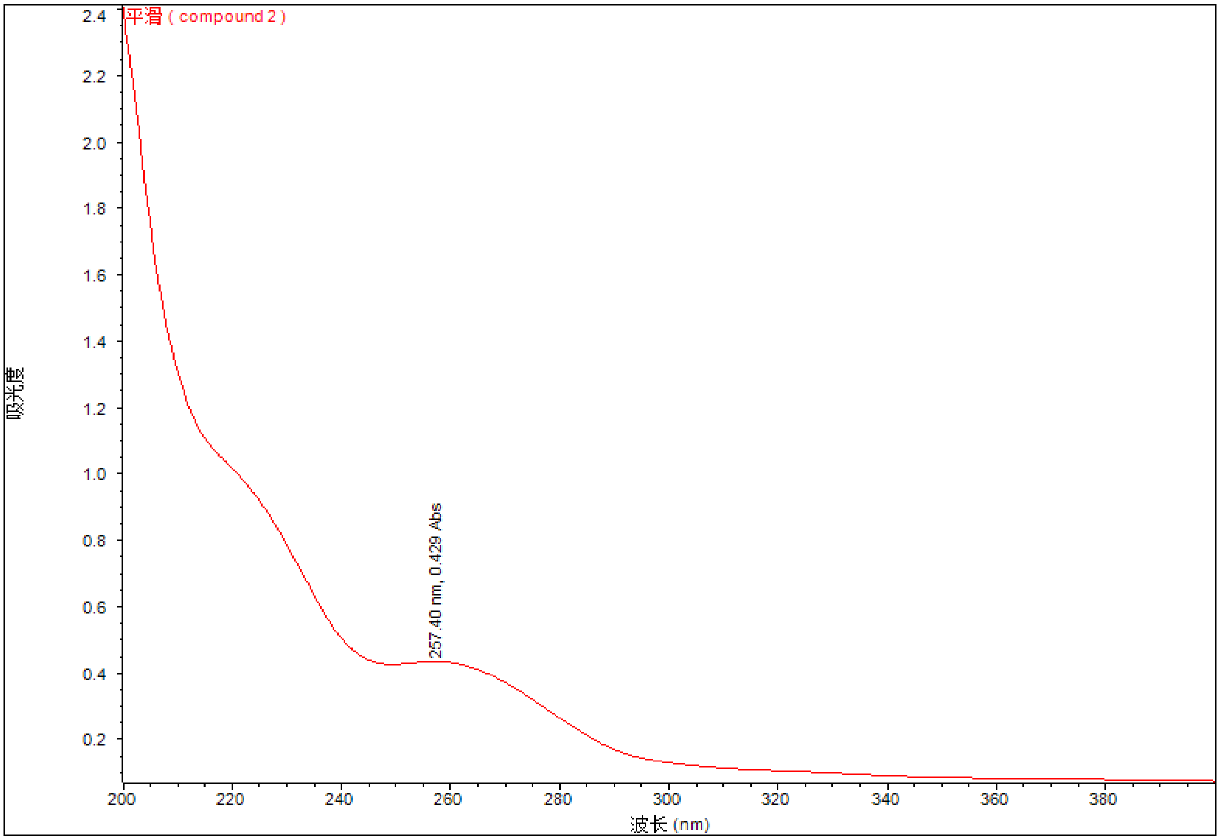

Figure S2d. UV spectrum of compound **2**

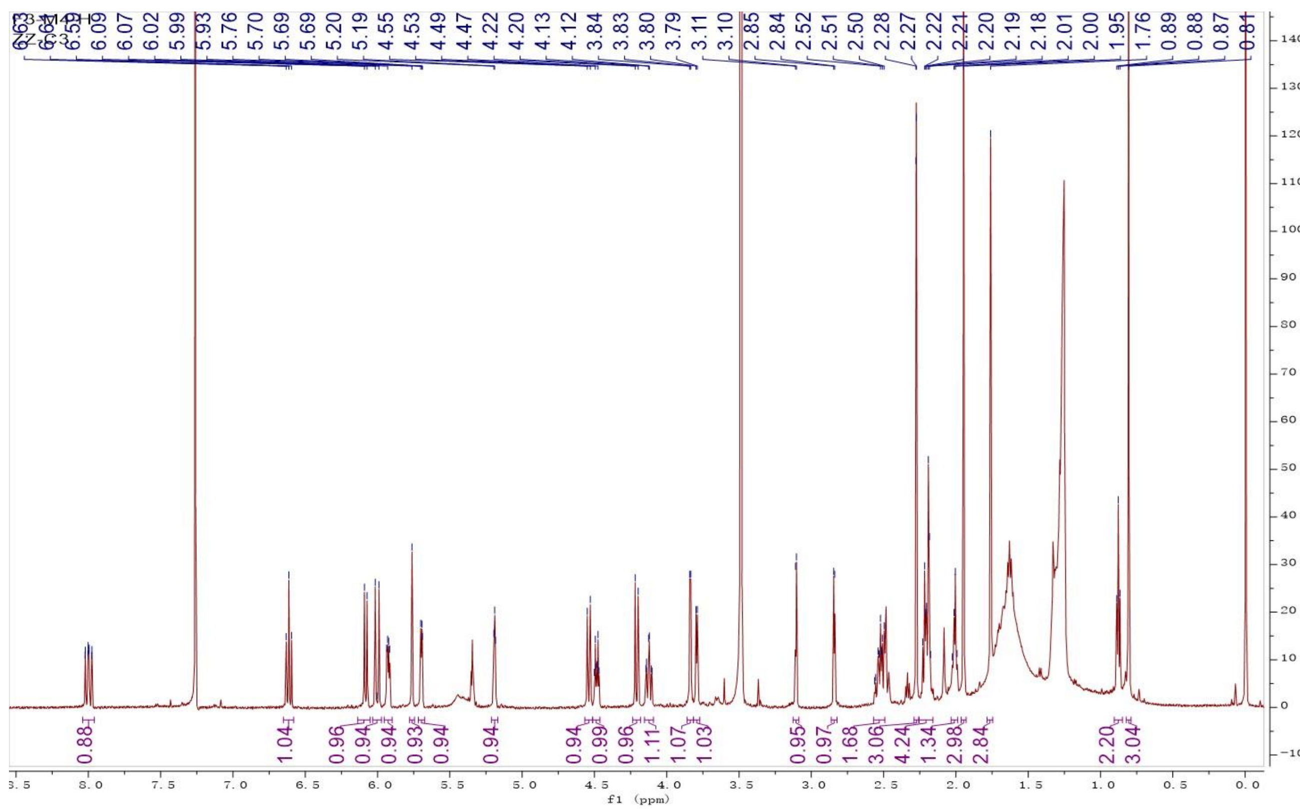

Figure S3a. ^{1}H NMR spectrum of compound **3** ($CDCl_3$, 600 MHz)

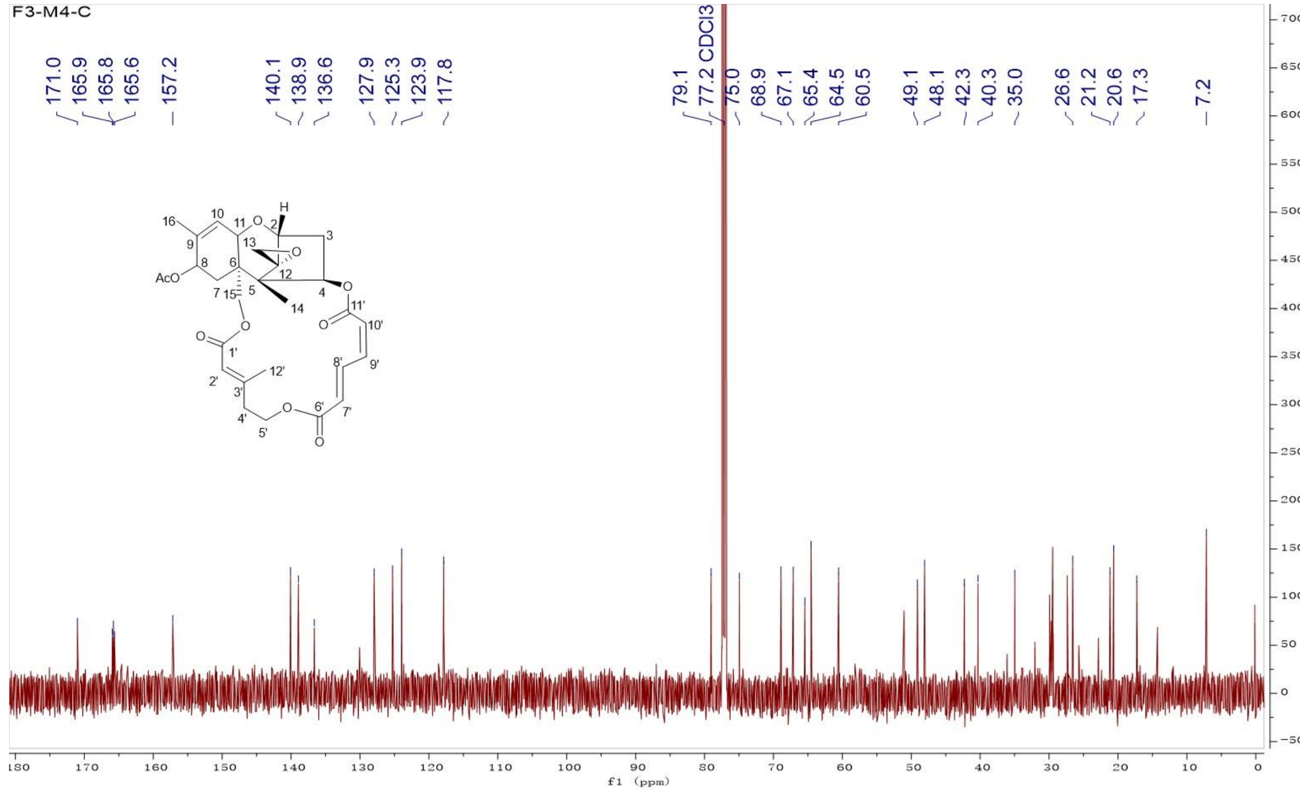

Figure S3b.^{13}CNMR spectrum of compound **3** ($CDCl_3$, 150 MHz)

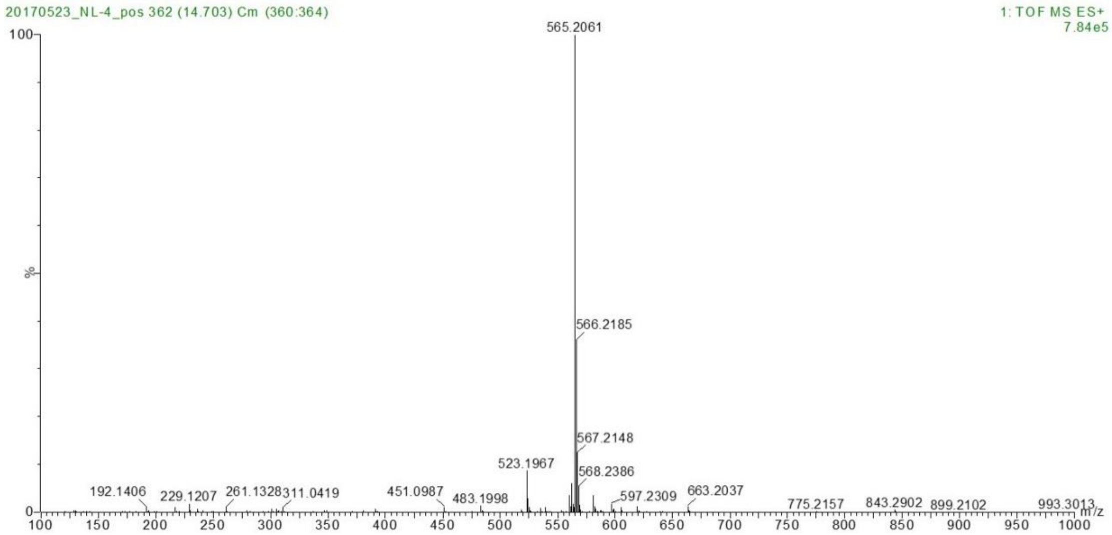

Figure S3c. HRESIMS spectrum of compound **3**

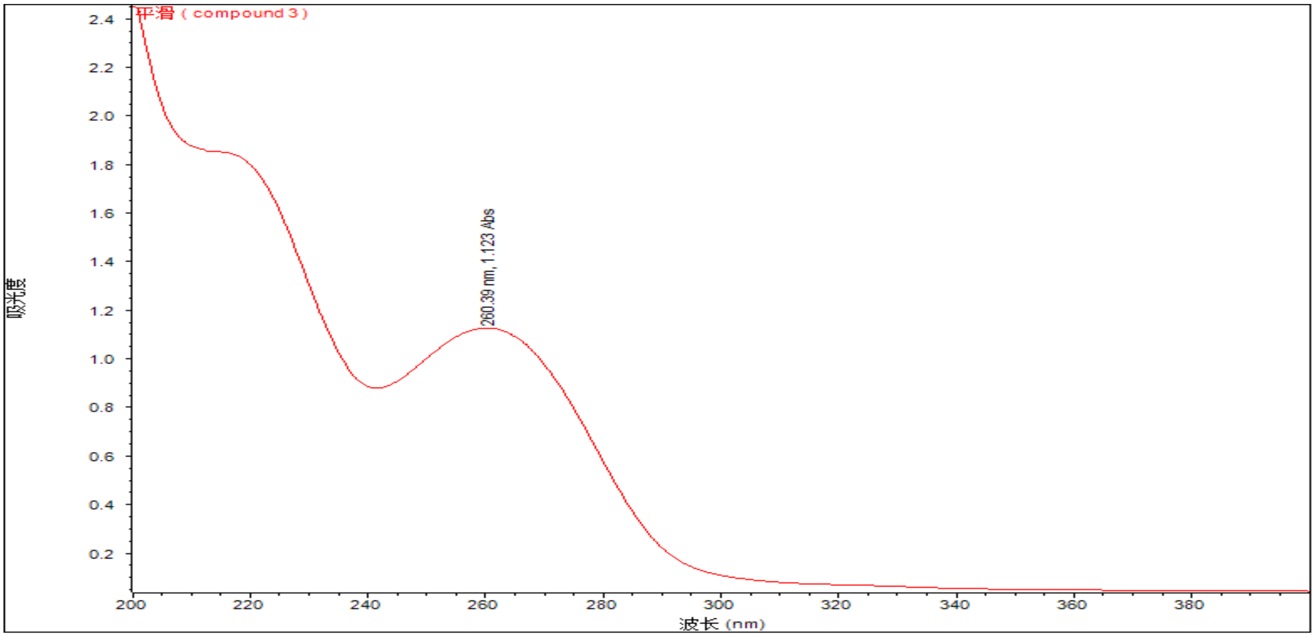

Figure S3d. UV spectrum of compound **3**

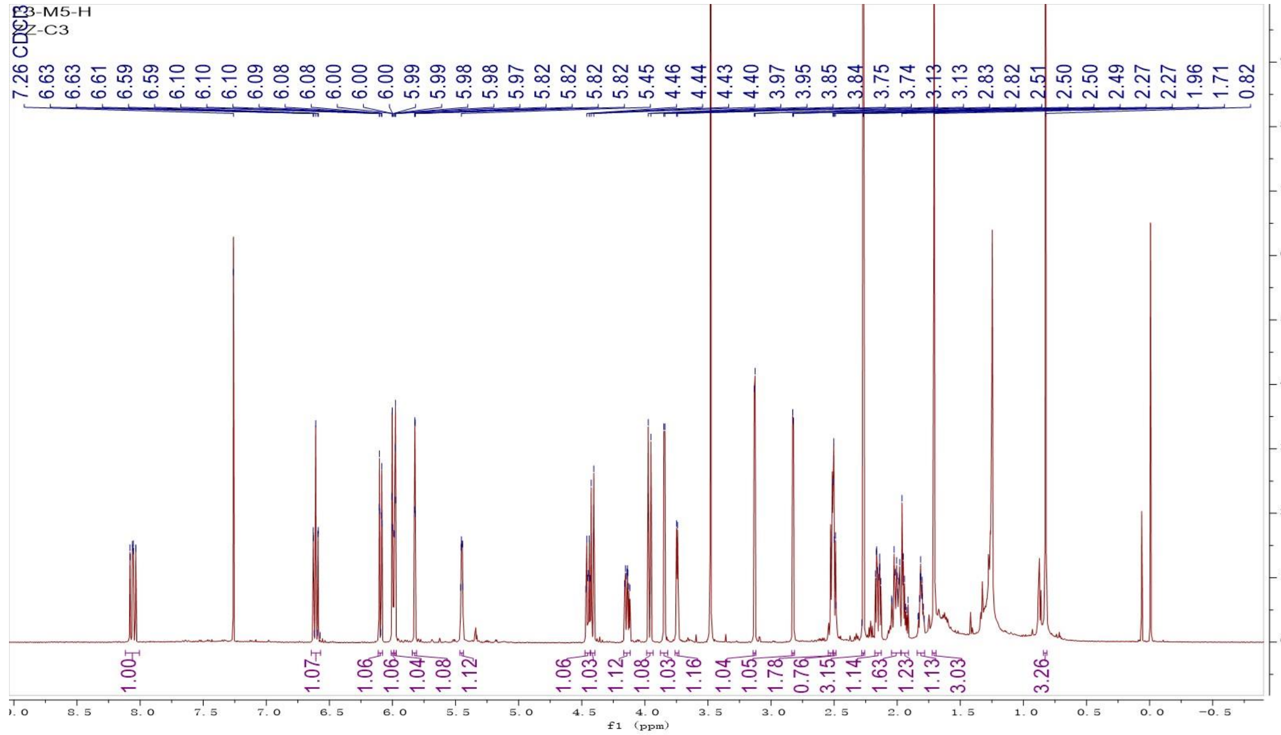

Figure S4a. ^{1}H NMR spectrum of compound **4** ($CDCl_3$, 600 MHz)

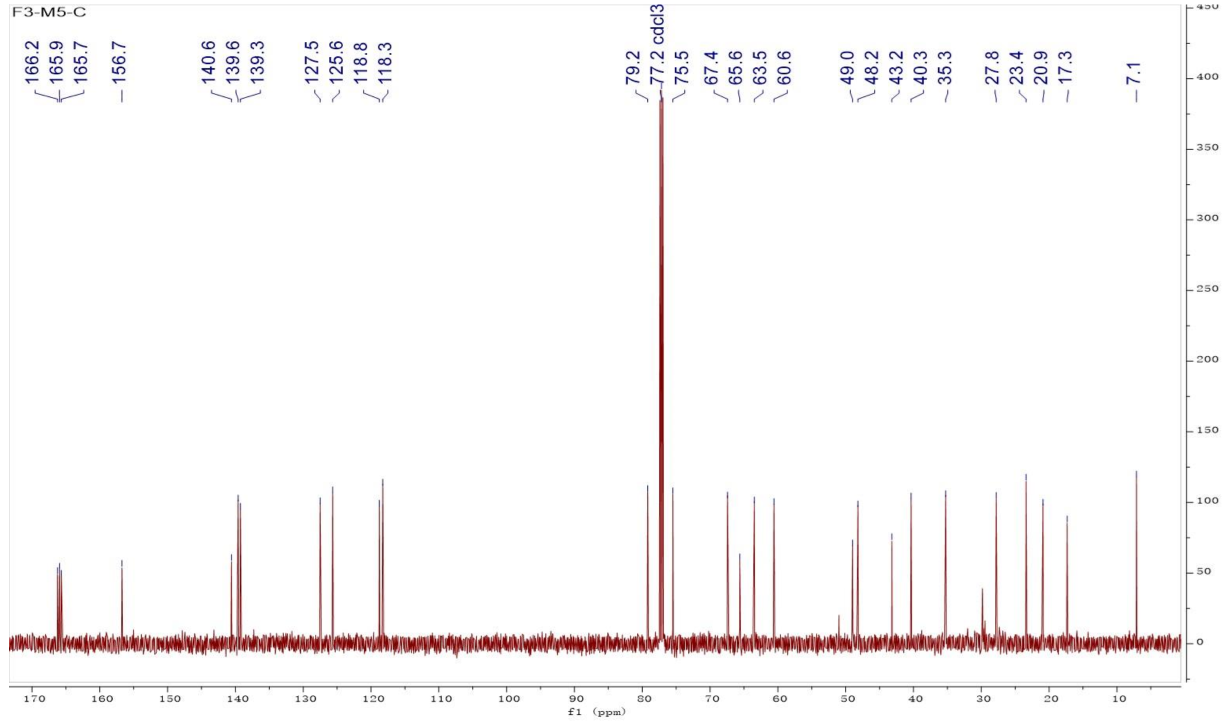

Figure S4b. ^{13}CNMR spectrum of compound **4** ($CDCl_3$, 150 MHz)

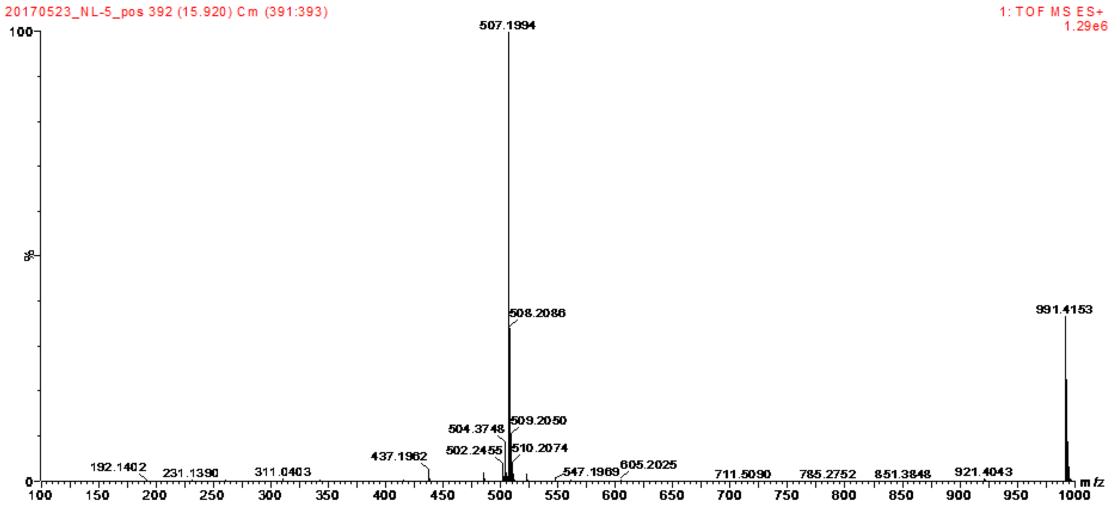

Figure S4c. HRESIMS spectrum of compound **4**

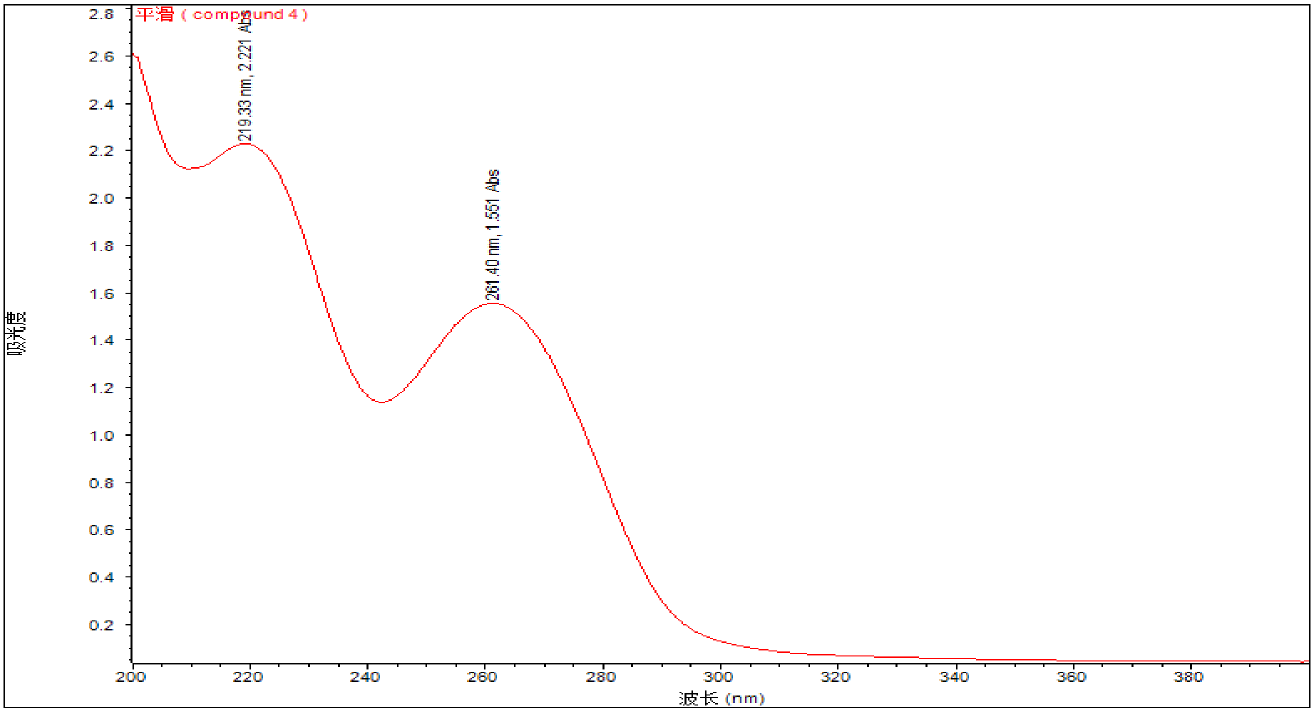

Figure S4d. UV spectrum of compound **4**

Table S1. NMR data of compound **1** in $CDCl_3$

Position	literature data (^{1}**H 600 MHz**, ^{13}**C 150 MHz**)[1]		experimental data(^{1}**H 600 MHz**, ^{13}**C 150 MHz)**	
	δ_H (mult, *J*)	δ_C	δ_H (mult, *J*)	δ_C
2	3.83 (1H, d, 5.0 Hz)	79.5	3.83 (1H, d, 5.1 Hz)	79.4
3	2.51 (1H, dd, 15.0, 8.0 Hz); 2.04 (1H, ddd, 15.0, 5.0, 4.0 Hz)	36.1	2.50 (1H,m); 2.05 (1H, m)	36.0
4	6.20 (1H, dd, 8.0, 4.0 Hz)	74.4	6.21 (1H, dd, 8.1, 3.9 Hz)	74.4
5		48.6		48.6
6		42.9		42.9
7	2.02 (1H, ddd, 12.6, 12.6, 5.5 Hz) 1.65 (1H, ddd, 12.6, 5.5, 1.4 Hz)	21.8	2.03 (1H, m)； 1.65 (1H, m)	21.8
8	2.01 (1H, ddd, 12.6, 12.6, 5.5 Hz); 2.00 (1H, ddd, 12.6, 5.5, 1.4 Hz)	27.9	2.00 (2H, m)	27.8
9		140.4		140.4
10	5.46 (1H, brd)	119.1	5.48 (1H, m)	119.0
11	3.89 (1H, brd)	67.5	3.90 (1H, d, 5.7 Hz)	67.4
12		65.8		65.8
13	3.12 (1H, d, 4.1 Hz); 2.80 (1H, d, 4.1 Hz)	48.4	3.14 (1H, d, 4.1 Hz); 2.82 (1H, d, 4.0 Hz)	48.3
14	0.77 (3H, s)	6.9	0.79 (3H, s)	6.8
15	4.29 (1H, d, 12.5 Hz); 3.92 (1H, d, 12.5 Hz)	64.0	4.31 (1H, d, 12.5 Hz); 3.93 (1H, d, 12.5 Hz)	63.9
16	1.69 (3H, brs)	23.5	1.71 (3H, m)	23.4
1′		166.7		166.6
2′	5.94 (1H, brs)	117.4	5.90 (1H, dd, 15.7, 3.4 Hz)	117.3
3′		159.4		159.4
4′	2.51 (1H, dd, 13.0, 7.3, ~1.0 Hz); 2.48 (1H, dd, 13.0, 6.8, <1.0 Hz)	41.5	2.51 (2H, m)	41.4
5′	3.62 (1H, ddd, 15.1, 6.8, ~1.0 Hz); 3.54 (1H, ddd, 15.1, 7.3, <1 Hz)	70.1	3.62 (1H, m); 3.56 (1H, dt, 8.4, 7.1 Hz)	70.0
6′	3.68 (1H, br,ddq, 6.7, 3.2, 1.0 Hz)	84.2		84.1
7′	5.88 (1H, dd, 15.7, 3.2 Hz)	138.2	5.96 (1H, d, 1.6 Hz)	138.1
8′	7.51 (1H, br.dd, 15.7, 11.3 Hz)	126.8	7.52 (1H, ddt, 15.8, 11.4, 1.5 Hz)	126.7
9′	6.55 (1H, t, 11.3 Hz)	143.9	6.57 (1H, t, 11.3 Hz)	143.8
10′	5.73 (1H, d, 11.3 Hz)	118.1	5.75 (1H, dd, 11.2, Hz)	118.0
11′		166.0		166.0
12′	2.24 (3H, d, 1.1 Hz)	20.6	2.26 (3H, d, 1.3 Hz)	20.5
13′	3.63 (1H, dq, 6.7, 6.2 Hz)	70.9	3.65 (1H, m)	70.8
14′	1.18 (3H, d, 6.2 Hz)	18.5	1.20 (3H, d, 6.3 Hz)	18.4
OH-13′	2.62		2.67	

Table S2. NMR data of compound **2** in $CDCl_3$

Position	literature data (^{1}**H 400 MHz**, ^{13}**C 100 MHz)**[2]		experimental data (^{1}**H 600 MHz**, ^{13}**C 150 MHz)**	
	δ_H (mult, *J*)	δ_C	δ_H (mult, *J*)	δ_C
2	3.86-3.84 (1H, m,)	79.3	3.84 (1H, d, 5.2 Hz)	79.3
3	2.53-2.45(1H, m); 2.09 (1H, m)	35.7	2.52 (1H, m)	35.7
4	6.13 (1H, dd, 8.1, 4.0 Hz)	74.3	6.13 (1H, dd, 8.2, 4.1 Hz)	74.4
5		48.6		48.7
6		42.9		43.0
7	2.05-1.98 (1H, m); 1.71 (1H, m)	21.4	2.03-1.98 (1H, m); 1.72 (1H, m)	21.4
8	2.05-1.98 (2H, m)	27.8	2.03-1.98 (3H, m)	27.8
9		140.3		140.3
10	5.47 (1H, d, 5.3 Hz)	119.0	5.47 (1H, dq, 5.6, 1.5 Hz)	119.0
11	3.86-3.84 (1H, m,)	67.4	3.85 (1H, d, 5.8 Hz)	67.4
12		65.7		65.7
13	3.13 (1H, d, 4.0 Hz); 2.82 (1H, d, 4.0 Hz)	48.2	3.13 (1H, d, 4.1 Hz); 2.81 (1H, d, 4.1 Hz)	48.2
14	0.80 (3H, s)	6.8	0.80 (3H, s)	6.9
15	4.35 (1H, d, 12.6 Hz); 3.95 (1H, d, 12.6 Hz)	63.6	4.34 (1H, d, 12.6 Hz); 3.95 (1H, d, 12.6 Hz)	63.6
16	1.68 (3H, brs)	23.3	1.70 (3H, s)	23.4
1′		166.1		166.1
2′	5.90 (1H, s)	117.3	5.89 (1H, d, 1.7 Hz)	117.3
3′		159.1		159.2
4′	2.53-2.45 (2H, m)	41.5	2.46 (2H, m)	41.6
5′	3.74 (1H, m); 3.53 (1H, m)	69.0	3.73 (1H, m); 3.53 (1H, ddd, 8.5, 5.9, 6.8 Hz)	69.1
6′		79.2		69.0
7′	5.88 (1H, m)	137.5	5.89 (1H, d, 1.7 Hz)	137.5
8′	7.50 (1H, m)	127.4	7.49 (1H, dddd, 15.7, 11.4, 2.0, 1.0 Hz)	127.4
9′	6.58 (1H, dd, 11.4, 11.3 Hz)	143.5	6.57 (1H, t, 11.3 Hz)	143.5
10′	5.75 (1H, d, 11.0 Hz)	117.9	5.74 (1H, dd, 11.2, 0.9 Hz)	117.9
11′		166.6		166.6
12′	2.26 (3H, s)	19.5	2.26 (3H, d, 1.3 Hz)	19.6
13′	5.12 (1H, qd, 6.3, 4.9 Hz)	70.6	5.11 (1H, qd, 6.4, 4.7 Hz)	70.6
14′	1.16 (3H, d, 6.5 Hz)	14.8	1.15 (3H, d, 6.4 Hz)	14.8
15′		170.4		170.5
16′	2.05 (3H, s)	21.3	2.04 (3H, s)	21.5

Table S3. NMR data of compound **3** in $CDCl_3$

Position	literature data (^{1}**H 400 MHz**, ^{13}**C 100 MHz)**[3]		experimental data (^{1}**H 600 MHz**, ^{13}**C 150 MHz)**	
	δ_H (mult, *J*)	δ_C	δ_H (mult, *J*)	δ_C
2	3.83 (1H, d, 5.0 Hz)	78.9	3.84 (1H, d, 5.1 Hz)	79.1
3	2.21 (1H, m); 2.49 (1H, m)	34.9	2,21 (1H, m); 2.48 (1H, m)	35.0
4	5.93 (1H, dd, 8.0, 4.0 Hz)	74.8	5.93 (1H, dd, 8.4, 4.3 Hz)	75.0
5		49.0		49.1
6		42.2		42.3
7	2.19 (2H, m)	26.5	2.19 (2H, d, 4.5 Hz)	26.6
8	5.19 (1H, m)	68.8	5.19 (1H, t, 3.2 Hz)	68.9
9		136.5		136.6
10	5.70 (1H, brd, 5.5 Hz)	123.8	5.70 (1H, d, 5.4 Hz)	123.9
11	3.80 (1H, d, 5.5 Hz)	67.0	3.84 (1H, d, 5.1 Hz)	67.1
12		65.3		65.4
13	2.84 (1H, d, 4.0 Hz);<br>3.11 (1H, d, 4.0 Hz)	47.9	2.84 (1H, d, 4.0 Hz);<br>3.11 (1H, d, 4.0 Hz)	48.1
14	0.81 (3H, s)	7.0	0.81 (3H, s)	7.2
15	4.21 (1H, d, 12.5 Hz);<br>4.54 (1H, d, 12.5 Hz)	64.4	4.21 (1H, d, 12.5 Hz);<br>4.54 (1H, d, 12.5 Hz)	64.5
16	1.76 (3H, s)	20.5	1.76 (3H, s)	20.6
17		170.9		171.0
18	1.95 (3H, s)	21.0	1.95 (3H, s)	21.2
1′		165.6		165.8
2′	5.77 (1H, brs)	117.7	5.76 (1H, s)	117.8
3′		157.0		157.2
4′	2.54 (2H, m)	40.2	2.52 (2H, m)	40.3
5′	4.13 (1H, ddd, 11.0, 11.0, 3.5 Hz)<br>4.49 (1H, ddd, 11.0, 4.0,4.0 Hz)	60.4	4.12 (1H, td, 11.2, 3.4 Hz)<br>4.48 (1H, dt, 11.4, 4.0,4.2 Hz)	60.5
6′		165.5		165.6
7′	6.01 (1H, d, 15.5 Hz)	127.8	6.00 (1H, d, 15.7 Hz)	127.9
8′	8.00 (1H, dd, 15.5, 11.5 Hz)	138.8	8.00 (1H, ddd, 15.5, 11.5, 1.1 Hz)	138.9
9′	6.62 (1H, dd, 11.5, 10.5 Hz)	139.9	6.61 (1H, t, 11.3 Hz)	140.1
10′	6.09 (1H, d, 10.5 Hz)	125.1	6.08 (1H, d, 11.1 Hz)	125.3
11′		165.8		165.9
12′	2.28 (3H, d, 1.0 Hz)	17.1	2.27 (3H, d, 1.2 Hz)	17.3

Table S4. NMR data of compound **4** in $CDCl_3$

Position	literature data (^{1}**H 400 MHz**, ^{13}**C 100 MHz**)[3]		experimental data (^{1}**H 600 MHz**, ^{13}**C 150 MHz**)	
	δ_H (mult, *J*)	δ_C	δ_H (mult, *J*)	δ_C
2	3.88 (1H, d, 5.0 Hz)	79.0	3.84 (1H, d, 5.1 Hz)	79.2
3	2.16 (1H, m); 2.45 (1H, m)	35.1	2.15 (1H, ddd, 15.3, 5.2, 4.2 Hz); 2.50 (1H, m)	35.3
4	6.00 (1H, m)	75.3	6.00 (1H, m)	75.5
5		48.8		49.0
6		43.0		43.2
7	1.84 (1H, m) 1.98 (1H, m)	20.8	1.81 (1H, m); 1.94 (1H, m)	20.9
8	2.00 (2H, m)	27.7	2.01 (2H, m)	27.8
9		140.4		140.6
10	5.28 (1H, brd, 5.0 Hz)	118.6	5.45 (1H, dq, 5.5, 3.3, 1.5 Hz)	118.3
11	3.68 (1H, d, 5.5 Hz)	67.3	3.74 (1H, d, 5.5 Hz)	67.4
12		65.5		65.6
13	2.83 (1H, d, 4.0 Hz); 3.12 (1H, d, 4.0 Hz)	48.1	2.82 (1H, d, 4.0 Hz); 3.13 (1H, d, 4.0 Hz)	48.2
14	0.83 (3H, s)	7.00	0.82 (3H, s)	7.1
15	3.98 (1H, d, 12.5 Hz); 4.42 (1H, d, 12.5 Hz)	63.3	3.96 (1H, d, 12.6 Hz); 4.41 (1H, d, 12.6 Hz)	63.5
16	1.72 (3H, s)	23.3	1.71 (3H, s)	23.4
1′		166.1		166.2
2′	5.83 (1H, s)	118.1	5.82 (1H, q, 1.2 Hz)	118.8
3′		156.6		156.7
4′	2.53 (2H, m)	40.2	2.51 (2H, m)	40.3
5′	4.15 (1H, m); 4.44 (1H, m)	60.4	4.14 (1H, ddd, 11.3, 9.0, 4.3 Hz); 4.45 (1H, dt, 11.3, 4.6 Hz)	60.6
6′		165.5		165.9
7′	6.02 (1H, d, 15.5 Hz)	127.4	6.09 (1H, dt, 11.1, 0.9 Hz)	127.5
8′	8.09 (1H, dd, 15.5, 11.2 Hz)	139.1	8.06 (1H, ddd, 15.7, 11.6, 1.1 Hz)	139.3
9′	6.62 (1H, dd, 11.2, 11.0 Hz)	139.5	6.60 (1H, m)	139.6
10′	6.10 (1H, d, 11.0 Hz)	125.5	6.09 (1H, dt, 11.1, 0.9 Hz)	125.6
11′		165.8		165.9.
12′	2.28 (3H, d, 1.5 Hz)	17.2	2.27 (3H, d, 1.3 Hz)	17.3

Table S5. Specific rotation values of compounds **1–4**.

compound	literature data	experimental data
**1**	$[\alpha]_D^{22.5}$ -22 (*c* 0.3, $CHCl_3$)[5]	$[\alpha]_D^{21}$ -25 (*c* 0.3, $CHCl_3$)
**2**	$[\alpha]_D^{23}$ +50 (*c* 0.85, $CHCl_3$)[2]	$[\alpha]_D^{21}$+52.5 (*c* 0.25, $CHCl_3$)
**3**	$[\alpha]_D^{27}$ +29.7 (*c* 0.52, $CHCl_3$)[4]	$[\alpha]_D^{22}$ +28 (*c* 0.3, $CHCl_3$)
**4**	$[\alpha]_D^{21}$ +8.1 (*c* 0.2, $CHCl_3$)[5]	$[\alpha]_D^{21}$ +6.7 (*c* 0.1, $CHCl_3$)

Table S6. UV values of compounds **1–4**.

compound	literature data (λ_{max} MeOH)	experimental data(λ_{max} MeOH)
1	263[5]	258
**2**	262[2]	257
**3**	262[3]	260
**4**	263[3]	261

References

1. Ridge, C. D.; Mazzola, E. P.; Colesb, M. P.;. Hinkley, S. F. R. Isolation and characterization of roridin E. *Magn. Reson. Chem.* **2016**, *55*, 337–340.
2. Isaka, M.; Punya, J.; Lertwerawat, Y.; Tanticharoen, M.; Thebtaranonth, Y. Antimalarial activity of macrocyclic trichothecenes isolated from the fungus *Myrothecium verrucaria*. *J. Nat. Prod.* **1999**. *62*, 329–331.
3. Namikoshi, M.; Kobayashi, H.; Yoshimoto, T.; Meguro, S.; Akano, K. Isolation and characterization of bioactive metabolites from marine-derived filamentous fungi collected from tropical and sub-tropical coral reefs. *Chem. Pharm. Bull.* **2000**, *48*, 1452–1457.
4. Jarvis, B. B.; Midiwo, J. O.; Desilva, T.; Mazzola, E. P. Verrucarin L, a new macrocyclic trichothecene. *J. Antibiot*. **1981**, *34*, 120–121.
5. Saikawa, Y.; Okamoto, H.; Inui, T.; Makabe, M.; Okuno, T.; Suda, T.; Hashimoto, T.; Nakata, M. Toxic principles of a poisonous mushroom *podostroma cornu-damae*. *Tetrahedron* **2001**, *57*, 8277–8281.
